# Real-World Evidence on the Effectiveness of Plexiglass Barriers in Reducing Aerosol Exposure

**DOI:** 10.20411/pai.v7i2.533

**Published:** 2022-11-04

**Authors:** Jennifer L. Cadnum, Annette L. Jencson, Samir Memic, Andrew O. Osborne, Maria M. Torres-Teran, Brigid M. Wilson, Abhishek Deshpande, Curtis J. Donskey

**Affiliations:** 1 Research Service, Louis Stokes Cleveland VA Medical Center, Cleveland, Ohio; 2 Case Western Reserve University School of Medicine, Cleveland, Ohio; 3 Geriatric Research, Education, and Clinical Center, Louis Stokes Cleveland VA Medical Center, Cleveland, Ohio; 4 Center for Value-Based Care Research, Cleveland Clinic Lerner College of Medicine, Cleveland, Ohio

**Keywords:** barriers, transmission, aerosol, ventilation, SARS-CoV-2

## Abstract

**Background:**

Barriers are commonly installed in workplace situations where physical distancing cannot be maintained to reduce the risk for transmission of respiratory viruses. Although some types of barriers have been shown to reduce exposure to aerosols in laboratory-based testing, limited information is available on the efficacy of barriers in real-world settings.

**Methods:**

In an acute care hospital, we tested the effectiveness of in-use plexiglass barriers in reducing exposure of staff to aerosolized particles. A nebulizer was used to release 5% NaCl aerosol 1 meter from staff members with and without the barrier positioned between the point of aerosol release and the hospital staff. Particle counts on the staff side of the barrier were measured using a 6-channel particle counter. A condensed moisture (fog) generating device was used to visualize the airflow patterns.

**Results:**

Of 13 in-use barriers tested, 6 (46%) significantly reduced aerosol particle counts detected behind the barrier, 6 (46%) reduced particle counts to a modest, non-significant degree, and 1 (8%) significantly increased particle counts behind the barrier. Condensed moisture fog accumulated in the area where staff were seated behind the barrier that increased particle exposure, but not behind the other barriers. After repositioning the ineffective barrier, the condensed moisture fog no longer accumulated behind the barrier and aerosol exposure was reduced.

**Conclusion:**

In real-world settings, plexiglass barriers vary widely in effectiveness in reducing staff exposure to aerosols, and some barriers may increase risk for exposure if not positioned correctly. Devices that visualize airflow patterns may be useful as simple tools to assess barriers.

**Figure:**
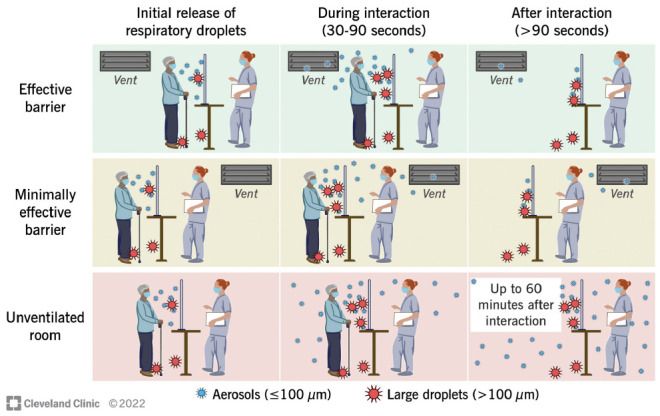
*Reprinted with permission, Cleveland Clinic Foundation ©2022. All Rights Reserved.*

## INTRODUCTION

Workers in healthcare and non-healthcare settings are at risk of acquiring infection with severe acute respiratory syndrome coronavirus 2 (SARS-CoV-2) and other respiratory viruses [1–5]. In work situations where physical distancing cannot be maintained, barriers are often installed to reduce the risk for viral transmission [6–8]. In laboratory-based testing, some types of barriers have been shown to reduce exposure to aerosols [9, 10]. For example, we found that solid physical barriers were effective in reducing contamination with an aerosolized benign virus, water soluble dye, or fluorescent microspheres in laboratory simulations, but openings in the barriers resulted in loss of efficacy [9]. However, there is concern that barriers that are not carefully installed have the potential to hinder good ventilation resulting in increased aerosol exposure [1, 6]. To reduce the risk that barriers might hinder ventilation, the Centers for Disease Control and Prevention (CDC) recommends that airflow distribution testing with tracer “smoke” or handheld fog generators should be conducted [1]. It is suggested that if such testing identifies stagnant air pockets, the barrier should be redesigned or reoriented [1].

Although barriers have been evaluated in laboratory settings, limited information is available on their efficacy in real-world settings. Therefore, we examined the effectiveness of in-use plexiglass barriers in reducing exposure of hospital staff to aerosolized particles. Because ventilation could have an impact on the efficacy of barriers, we also conducted simulations to assess the effectiveness of a barrier in a non-ventilated room.

## METHODS

### Assessment of In-Use Barriers

In an acute care hospital, we tested the effectiveness of 13 in-use plexiglass barriers in reducing exposure of staff to aerosolized particles. The barriers ranged in size from width 76.2 cm x height 71.1 cm to width 76.2 cm x height 76.2 cm. All the barriers had an opening to pass items that ranged in size from 155.1 cm^2^ to 762.0 cm^2^.

A Preval sprayer (Nakoma Products, LLC) aerosol-based spray system was used to release 6 mL of 5% sodium chloride (NaCl) over 10 seconds; based on particle count readings, the device disperses predominantly 0.3 µm to 5-µm droplets (authors' unpublished data). In preliminary experiments, the impact of barriers was similar for the 10-second aerosol release with the Preval aerosol-based spray system and a 3-minute release of aerosol with a nebulizer (data not shown). The aerosol was released both with and without the barriers in place with 1 meter between the points of aerosol release and detection (30 cm from aerosol release point to the barrier plus 70 cm from barrier to position of the personnel). The aerosol was released at a height of 1.7 meters and directed toward the position where personnel would be seated behind the barrier. A 6-channel particle counter (Fluke 983, Fluke) was positioned in the location where personnel would be sitting at a height of 1.3 meters from the floor. The heights of the particle release and detection were chosen based on observations that patients usually stood behind the barriers while personnel were usually seated. Particle count readings of 1 μm- to 10 μm-sized particles were recorded for 2 minutes after particle release; particle counts consistently returned to baseline levels by 2 minutes after release in ventilated areas. The assessments were repeated in triplicate for each barrier, both with and without the barrier in place.

For each barrier, a condensed moisture (fog) airflow visualizer (CBreeze, Degree Controls, Inc.) was used to assess the direction of airflow with and without the barrier in place. The condensed moisture was released at the points where the aerosol was released in front of the barrier and at each side of the barrier. For those barriers that significantly increased personnel exposure to the NaCl aerosol, additional assessments of airflow were conducted using an anemometer (AN200, Extech Instruments) to measure airflow speed and direction with and without the barrier in place. In addition, for barriers that increased personnel aerosol exposure, we evaluated whether repositioning the barrier would reduce personnel exposure.

### Simulations to Assess Barrier Effectiveness in a Non-Ventilated Room

Ventilation systems create airflow patterns that can potentially facilitate transmission of respiratory viruses and that may impact the efficacy of barriers [1, 11, 12–15]. Because all the in-use barriers tested were in well-ventilated settings, we conducted additional simulations to assess the effectiveness of a barrier in a non-ventilated room (41.7 cubic meters). We hypothesized that barriers would be less effective in non-ventilated rooms because aerosol particles might disperse throughout the room and persist, thereby obviating the benefit that the barrier might provide in reducing initial exposure after aerosol is generated.

The efficacy of the barrier was tested as described previously. The aerosol was released both with and without a 60 cm x 76 cm barrier with no opening in place. A Preval sprayer aerosol-based spray system was used to release 6 mL of 5% NaCl over 10 seconds directed toward the barrier. Particle counts of 1 μm- to 10 μm-sized particles were recorded every 30 seconds for 2 minutes after aerosol release, every minute for 10 minutes after aerosol release, and at 20 and 30 minutes. The total distance between aerosol release and particle count sampling was 1 meter as described previously.

### Data Analysis

For in-use barriers, we compared the particle counts detected behind the barrier over 2 minutes after particle release with and without the barrier present. A 2-way analysis of variance (ANOVA) was performed, comparing with versus without the barrier present for each barrier. A square root transformation of accumulated particles was performed to meet model assumptions. Post-hoc contrasts within barriers were assessed. As we considered each barrier to be independent and wished to control type I error at the barrier level, no post-hoc correction was performed. For the simulations in the non-ventilated room, we compared the peak particle counts detected at 30 seconds after aerosol release and the cumulative particle count detected behind the barrier over 30 minutes with versus without the barrier present. Data were analyzed using R version 4.1.3 software (R Foundation for Statistical Computing).

## RESULTS

### In-Use Barriers

**Figure 1.**
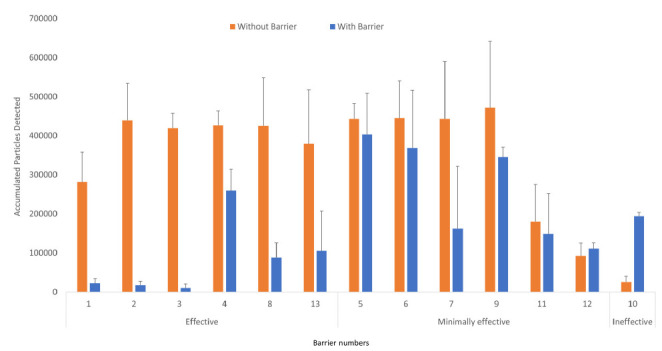
**Accumulated number of 5% sodium chloride aerosol particles detected over 2 minutes with versus without a plexiglass barrier in place.** The aerosol particles were released at 91 cm from the barrier. Average results for 3 experiments are shown. Error bars represent standard error. Effective barriers significantly reduced particle exposure; minimally effective barriers reduced exposure but not significantly; the ineffective barrier resulted in a significant increase in exposure.

**Figure 2.**
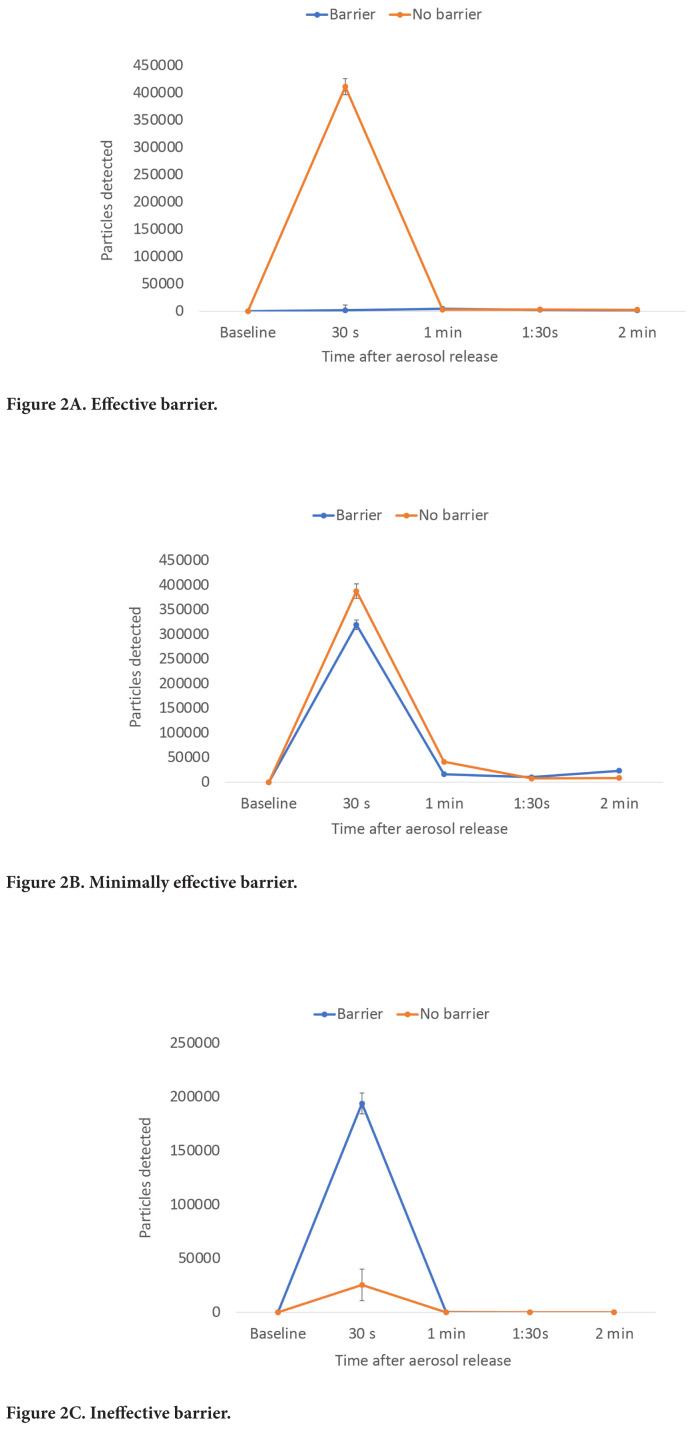
**Number of 5% sodium chloride aerosol particles detected over 2 minutes with versus without a plexiglass barrier in place for 3 typical barriers, including an effective, minimally effective, and ineffective barrier.** The aerosol particles were released at 91 cm from the barrier. Average results for 3 experiments are shown. Error bars represent standard error.

Of 13 in-use barriers tested, 6 (46%) significantly reduced aerosol particle counts detected behind the barrier, 6 (46%) reduced particle counts to a modest, non-significant degree, and 1 (8%) significantly increased particle counts behind the barrier (Figure 1). For all barriers, aerosol particle counts peaked at 30 seconds after release and rapidly decreased to low levels at or close to baseline by 1 to 2 minutes after release. Figure 2 shows aerosol particles detected over 2 minutes for 3 barriers, including the ineffective barrier and an effective and minimally effective barrier.

**Figure 3.**
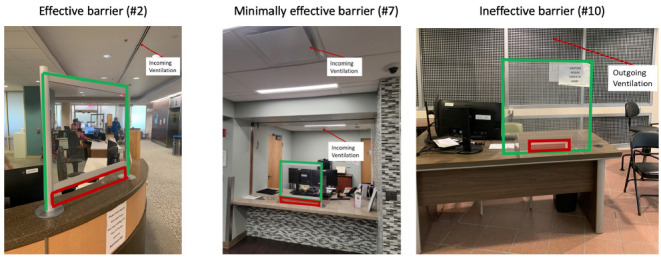
**Pictures of 3 of the barriers tested.** The red rectangles outline the location of openings of the barriers and the green lines outline the outer borders of the barriers. Barrier 2 is width 30” x height 28” with an opening of width 30” x height 4”. Barriers 7 and 10 are width 30” x height 30” with an opening of width 12” x height 2”.

For the 6 barriers that significantly reduced aerosol particle exposure, the closest incoming or outgoing air vents were positioned on the ceiling on the patient side. For the 6 barriers that reduced particle counts only modestly, the closest air vents were positioned on the personnel side of the barrier. Condensed moisture fog released on the patient side initially flowed toward the nearest outgoing or incoming vent (ie, incoming air vents produced currents that initially pulled air toward the vent with subsequent movement to outgoing vents). Figure 3 provides pictures of 3 of the barriers, including an effective, minimally effective, and ineffective barrier.

The ineffective barrier that increased personnel exposure to particles had a unique ventilation system with a large outgoing air vent positioned behind the barrier on the side wall; all other outgoing vents were positioned on the ceiling. Without the barrier present, the accumulated particles detected behind this barrier were substantially lower than for the other settings without the barrier, potentially due to the unique outgoing vent with rapid outflow of aerosol particles. With the barrier in place, the condensed moisture fog flowed around the sides of the barrier and then into the space immediately behind the barrier. After pooling behind the barrier, the condensed moisture fog flowed to the outlet air vent positioned on the side wall.

**Figure 4.**
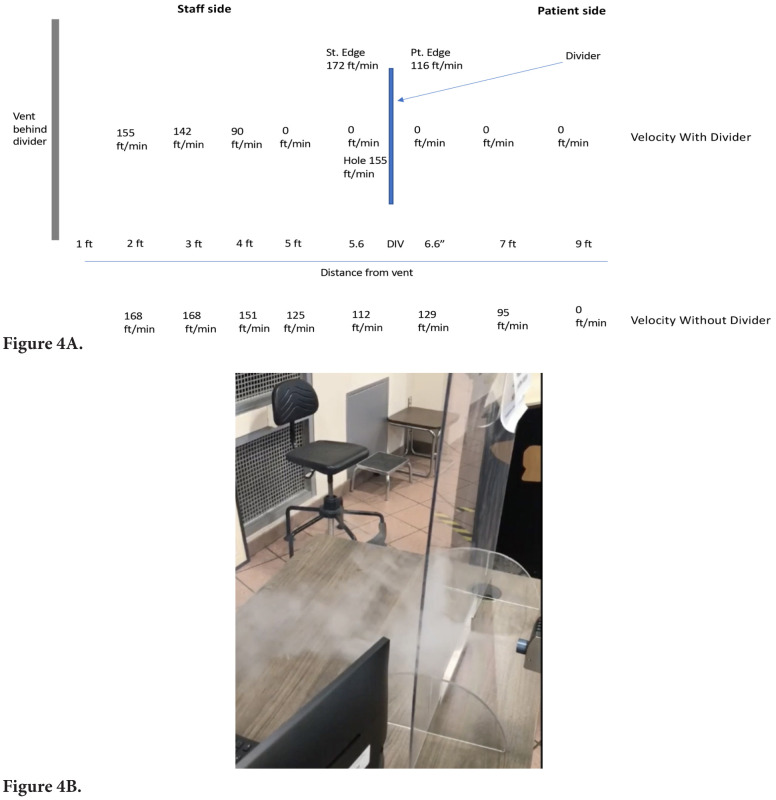
**Airflow readings with versus without the plexiglass barrier in place for the 1 barrier (#10) that resulted in increased aerosol particle exposure (A) and a video demonstrating movement of condensed moisture fog into the space immediately behind the ineffective barrier (B).** For the video illustration, the fog-generating device was placed closer to the edge of the barrier than the point of aerosol release because that provided a clearer illustration as the fog dissipated less than when released at a greater distance.

Anemometer readings demonstrated that the presence of the barrier resulted in an area of low airflow immediately behind the barrier with rapid airflow on each side of the barrier. Figure 4 shows the airflow readings with versus without the barrier in place and a video that demonstrates movement of the condensed moisture fog into the space immediately behind the barrier. Repositioning of the personnel desk plus barrier 3 meters laterally resulted in the barrier being effective in reducing personnel exposure to aerosol particles.

### Simulations to Assess Barrier Effectiveness in a Non-Ventilated Room

**Figure 5.**
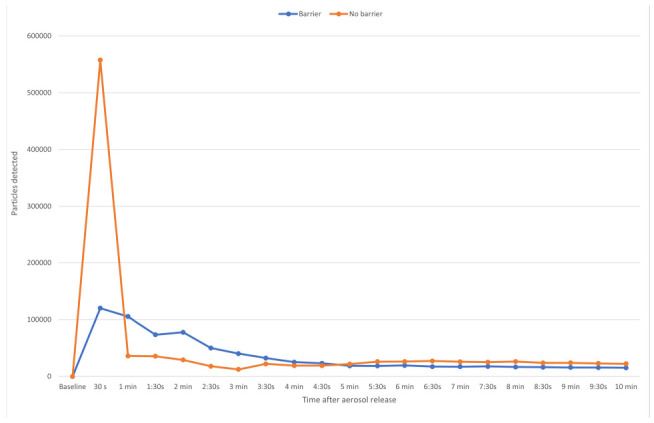
**Detection of 5% sodium chloride aerosol particles behind a plexiglass barrier over 30 minutes after release with versus without a plexiglass barrier in place in a non-ventilated room.** The aerosol particles were released at 91 cm from the barrier. Average results for 3 experiments are shown. Error bars represent standard error.

Figure 5 shows the number of particles detected in the non-ventilated room over 10 minutes both with and without the barrier in place. The barrier was effective in reducing initial peak exposure to aerosol particles (*P*<0.05). However, in the absence of ventilation, counts of aerosol particles remained elevated in the room throughout the 30-minute air sampling period, and total aerosol particle exposure over 30 minutes was not significantly different with versus without the barrier present (*P=*0.18). The aerosol particle counts were 30,082 at 6 minutes, 25,123 at 20 minutes, and 23,647 at 30 minutes.

## DISCUSSION

Although barriers are commonly installed in workplaces, there is controversy regarding their efficacy and concern that some barriers could be harmful due to disruption of normal ventilation [1]. However, little real-world evidence has been available on the efficacy of barriers. In the current study, we demonstrated that in-use plexiglass barriers in a hospital varied widely in effectiveness in reducing exposure of personnel to aerosols, and one barrier increased the risk for exposure.

Our results provide support for the CDC recommendation that devices that visualize airflow patterns should be used to assess barriers and guide repositioning if adverse effects on airflow are identified. No condensed moisture fog accumulated behind barriers that reduced aerosol particle exposure. However, for the one barrier that increased exposure to aerosol, condensed moisture fog testing demonstrated movement of air around the barrier and into the area where personnel were sitting. After repositioning the barrier, the condensed moisture fog no longer pooled behind the barrier and aerosol exposure was reduced. In general, positioning of vents in relationship to the barriers appeared to impact effectiveness (ie, for more effective barriers the closest incoming or outgoing air vents were positioned on the ceiling on the patient rather than personnel side). However, visualization of airflow patterns is needed given the wide variety of barrier and vent configurations in real-world settings.

The in-use barriers in our study were all in ventilated areas. The simulations completed in the non-ventilated room suggest that barriers may be much less effective in areas with poor ventilation. In the non-ventilated room, the barrier reduced initial aerosol particle exposure, but not subsequent exposure as the aerosol particles disseminated throughout the room.

In our previous laboratory study, we found that barriers with openings were significantly less effective in reducing contamination with an aerosolized benign virus, water soluble dye, or fluores-cent microspheres [9]. However, in the current study we found that several barriers with openings were effective in reducing exposure to aerosol particles. One potential explanation for the differing results is that the aerosol particles were released at a height of 1.7 meters in the current study based on observations that patients typically stood behind the barrier versus 1.3 meters in the previous study. The higher release point might have reduced the likelihood that aerosol particles would transfer through the openings, which were typically at waist height. Because all the in-use barriers had openings, we cannot exclude the possibility that barriers with no openings would have been more effective. However, there was no obvious correlation between the size of the opening and effectiveness of the in-use barriers. For the minimally effective and effective barriers, the average opening size was 226.52 cm^2^ (range, 60.96 cm^2^ and 304.8 cm^2^) and 424.18cm^2^ (range, 304.8 cm^2^ and 1,325.88 cm^2^). The opening of the ineffective barrier was 69.96 cm^2^.

Our study has some limitations. The assessments performed cannot replicate all factors that may affect barrier efficacy in real-world settings. The in-use barrier testing was conducted in one building with good ventilation and only 13 barriers were assessed. Additional testing is needed in community settings with suboptimal ventilation. We did not assess the efficacy of barriers in comparison to or in combination with measures such as facemasks, increased ventilation rates, and portable air cleaners [16]. Finally, the aerosol particles released by the Preval sprayer aerosol-based spray system may differ in number and characteristics from those released by people with viral respiratory infections. Previous studies have demonstrated that a cough produces approximately 3,000 droplets, a sneeze releases an estimated 40,000 droplets, and loud speaking may release thousands of droplets per second [17–19].
